# A rare cause of recurrent acute pancreatitis

**DOI:** 10.1002/jgh3.12391

**Published:** 2020-07-13

**Authors:** Ujjwal Sonika, Ashok Dalal, Ajay Kumar

**Affiliations:** ^1^ Department of Gastroenterology, Academic Block GB Pant Hospital New Delhi India

**Keywords:** annular pancreas, ERCP, pancreatic duct sphincterotomy, recurrent acute pancreatitis

## Abstract

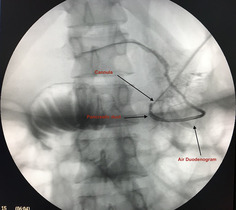

Annular pancreas is a rare entity. It clinically presents with intestinal obstruction. We present a case of a young female with incomplete annular pancreas presented with recurrent acute pancreatitis.

A 28‐year‐old woman presented to the Gastroenterology out ‐ patient department (OPD) with a history of recurrent episodes of mild pancreatitis for the last 2 years. Her physical examination was unremarkable. All the laboratory investigations were normal. The Magnetic Resonance Cholangio‐pancreatography (MRCP) report was suggestive of pancreatic divisum. The Endoscopic Retrograde Cholangio‐Pancreatography (ERCP) was performed, and pancreatogram showed the main pancreatic duct wrapping around the duodenum and going toward the spine (Fig. 1); suggestive of incomplete annular pancreas. A pancreatic duct (PD) sphincterotomy was done. The patient is on follow‐up for the last 6 months. She is asymptomatic until now with no new episodes of abdominal pain.

**Figure 1:**
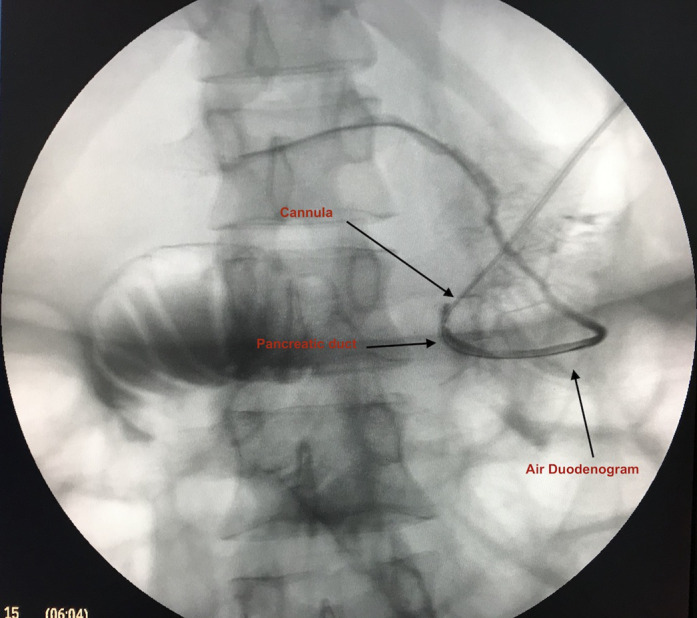
ERCP image showing the pancreatic duct wrapping around the duodenum (visible as air duodenogram) and going toward spine.

Annular pancreas is a rare congenital malformation with an estimated incidence of 5–15 cases per 100 000 adults.^1^ In this entity, a ring or annulus of pancreatic tissue is wrapped around the second part of pancreas. The majority of people remain asymptomatic. Although its more common clinical presentation is upper intestinal obstruction, it can also lead to recurrent acute pancreatitis.^2^, ^3^ In incomplete or partial annular pancreas, this annulus of pancreatic tissue does not surround the duodenum completely.^4^ The partial annular pancreas can also cause duodenal obstruction or pancreatitis.^5^ The endoscopic PD sphincterotomy, although not established as a measure to prevent recurrent acute pancreatitis in partial or complete annular pancreas, was effective in our case.
